# Evidence suggesting that microglia make amyloid from neuronally expressed APP: a hypothesis

**DOI:** 10.1186/s13024-025-00847-8

**Published:** 2025-05-09

**Authors:** John Hardy, Patrick Lewis

**Affiliations:** 1 Department of Neurodegenerative Disease, UCL Institute of Neurology, London, UK; 2 UK Dementia Research Institute at UCL, London, WC1N 3BG UK; 3 Hong Kong University of Science and Technology, Hong Kong, Hong Kong; 4 Department of Comparative Biomedical Sciences, Royal Veterinary College, University of London, London, NW1 0TU UK

## Abstract

While APP is largely neuonally expressed, Aβ amyloid is largely produced by microglia as the clearance mechanisms for damaged membranes becomes overwhelmed.

Over the last 10 years, genetic data coupled with functional analysis has increasingly pointed at the role of loss of microglial function as a key determinant for the risk of late onset Alzheimer’s disease [1]. This had been a surprise because the genetics of early onset disease indicated that neuronal APP processing underpinned early onset autosomal dominant disease [2]. We have reconciled these two contrasting etiologies by suggesting that production either of too much Aβ or production of a less soluble, longer Aβ underpinned early onset disease and that the risk of late onset disease was predisposed to by deficiencies in the (largely) microglial clearance of Aβ. In support of this idea, we adduced both transgenic APP mouse gene expression data and non-radioactive isotopic labelling experiments examining APP metabolism in cases and controls [3]. While these data convincingly implicate microglia in the Aβ response they do not explain the mechanism tying the microglial response to amyloid deposition.

British dementia, formerly known as Worster-Drought syndrome, is the other disease which is characterised by the occurrence of plaques and tangles. In this kindred, a nonsense codon mutation in the ITM2B gene leads to the production of the mutated ABri protein which has an additional 10 amino acids added to the to the C-terminal of the ITM2B protein. Furin cleavage of this nonsense sequence leads to the production of a novel 34 amino acid protein which deposits as neuritic amyloid plaques. Interestingly, these individuals also develop neurofibrillary tangles. It had always been assumed that these proteins were neuronally produced: indeed, attempts to model this disease used neuron specific promoters. However, recent data shows that this protein is almost exclusively produced by microglia [4]. Thus, in this plaque and tangle disease, the amyloid is almost certainly largely produced by microglia. The importance of this work is that it demonstrates not only that microglia can produce amyloid, but also that they do so in a fashion which leads to neuritic plaques and neurofibrillary tangles.

In electron microscopic studies both Gray, and colleagues [5] and Frackowiak and colleagues [6] examined Alzheimer brains and their amyloid production by electron microscopy (Fig. 1, reproduced from ref. 5). These papers both clearly show amyloid fibril production originating from coated pits in microglia.

**Fig. 1:**
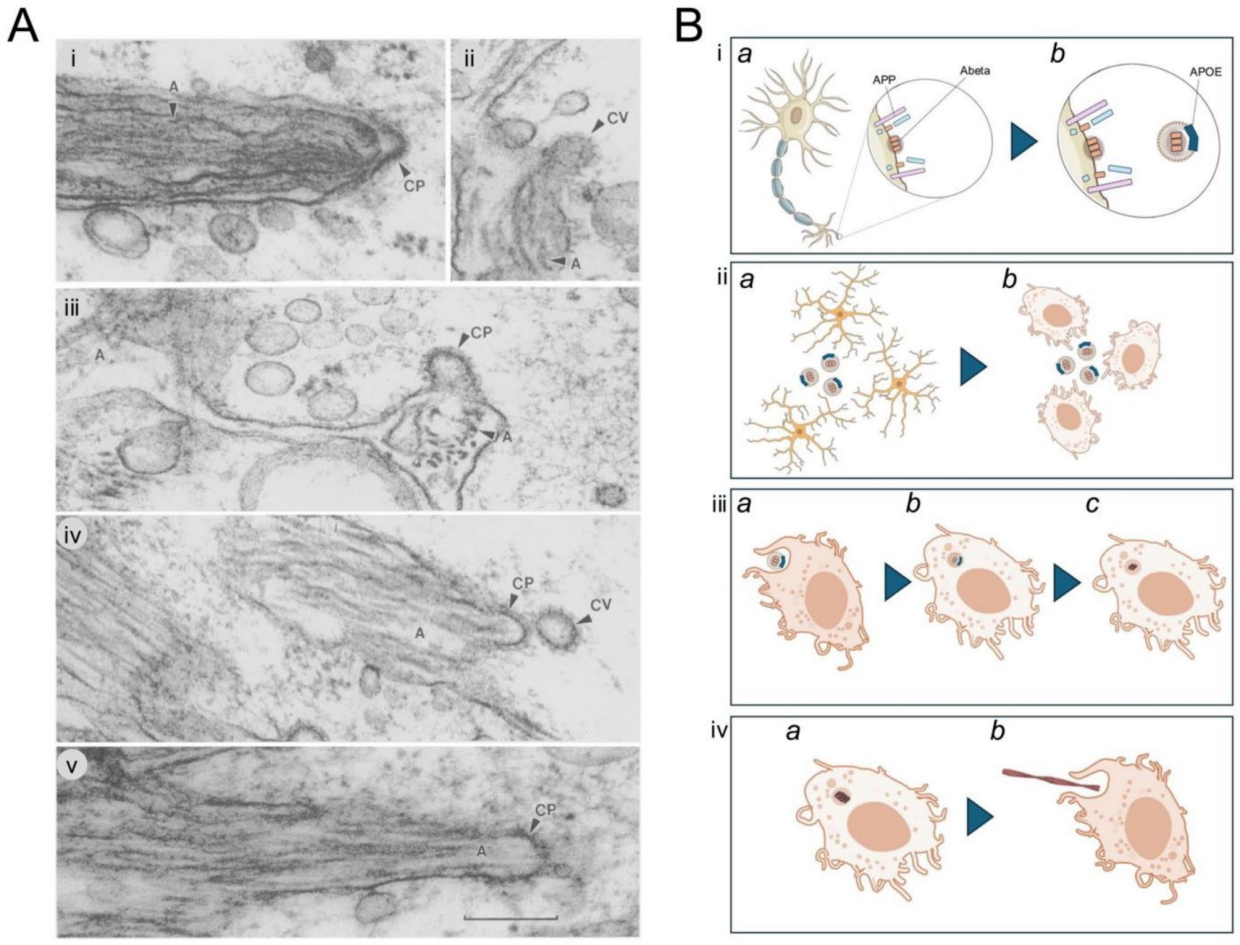
-Alzheimer amyloid fibrils (**A**) being extruded from microglial coated pits (CP). Image reproduced with permission from Ref 5. (**B**) (i) Aβ formed by APP metabolism intramembraneously largely at neuronal synapses. (ii): the Aβ loaded membrane fragments are picked up in apoe lipoproteins: these attract microglia, via their TREM2 receptors, which are then activated. (iii) fragments are taken up by phagocytosis and engulfed and then these lipoproteins are delipidated, and begin to seed larger aggregates. Finally, these enter the efferocytosis pathway (iv) and extrude amyloid fibrils

Most recently, Simmonds and colleagues suggested that interaction between Aβ and apoe happens in microglial lysosomes [7]. This builds on earlier work from Yeh and colleagues showing that uptake into microglia is TREM2 dependent [8], although the precise site of Aβ interaction with apoe remains unclear [9].

In addition, there is a considerable literature on the ageing of microglia, particularly from Streit and colleagues who argued that microglial ageing rather than amyloid deposition underpinned late onset Alzheimer pathogenesis [10]. In their pathogenic construct their (unsatisfactory) argument was that early onset disease, where amyloid production is clearly important, is simply a different disorder from the late onset disease. Additionally, Millet and colleagues [11] have demonstrated an exhausted microglial transcriptional profile in aged brains and apoe4 genotyped brains consistent with the view that microglial exhaustion was an important contributory factor to Alzheimer pathogenesis.

How can these disparate observations be reconciled into a single pathogenic synthesis accounting for both early onset disease where amyloid production is clearly implicated and late onset disease, where microglial failure seems to be the culprit?

We suggest the following sequence of events (illustrated in Figure) which we hope incorporates the primacy of the importance of amyloid production derived from the analysis of the early onset disease with the evidence of microglial exhaustion from the literature on aging. This view is largely consistent with the study of Jacquet ey al. [12] suggesting microglia were principally responsible for producing amyloid fibrils.

1. APP processing occurs in neuronal membranes generating Aβ stubs within these membranes. These are initially cleared in apoe lipoproteins, but with ageing and with other damage this clearance mechanism becomes overwhelmed and neuronal membrane disruption begins, causing exposure of phosphatidyl serine [13].
2. These attract microglia cells which become activated and adopt a phagocytic phenotype.
3. The activated microglia engulf the membrane fragments including the APP/Aβ stubs and process these via the microglial endosomal lysosome system where delipidation occurs.
4. Eventually however, the microglial endosome lysosome clearance system becomes overwhelmed and delipidation of the Aβ stubs initiates the process of amyloid fibril formation within the microglia. These are then extruded as amyloid by the microglia thus initiating plaque formation.

This scheme is consistent with a general view that, overall, Alzheimer’s disease is precipitated by a microglial overload and exhaustion as suggested by Streit and colleagues [10]. In individuals with APP duplications and point mutations and presenilin mutations, their microglia clearance mechanisms become overloaded at an earlier age because constitutive APP metabolism occurs at lipid membranes at synapses and leads to more lipophilic Aβ production. More generally in “typical” late onset disease the problem is age dependent, microglial exhaustion. Those with reduced function variants in this pathway (such as TREM2 R47H) reach this critical juncture at an earlier age.

Recent positive clinical trial data has clearly validated amyloid removal as a target for late onset Alzheimer’s disease. While this vindicates the amyloid hypothesis, to date only disease slowing rather than disease halting has been demonstrated. The underlying pathogenesis of the residual clinical decline in these trials is unclear, however mouse data suggesting that microglial paralysis [14] may facilitate tangle formation could indicate that microglial fatigue underlies part of this continuing decline and that efforts to understand and combat this exhaustion are worthwhile.
